# Semaglutide-Associated Acute Pancreatitis in a Patient With Type 2 Diabetes Mellitus: A Case Report

**DOI:** 10.7759/cureus.105591

**Published:** 2026-03-21

**Authors:** Piyush Puri, Michael Akhavan, Jonathan Shadan, Tzipora Levitt, Aakash A Soni

**Affiliations:** 1 Internal Medicine, Icahn School of Medicine at Mount Sinai, Queens Hospital Center, New York, USA; 2 Medicine, Icahn School of Medicine at Mount Sinai, Queens Hospital Center, New York, USA

**Keywords:** exogenous pancreatic insufficiency, glp-1 analogue, medication-induced pancreatitis, obesity, ozempic, pancreatitis, semaglutide

## Abstract

Semaglutide, a glucagon-like peptide-1 (GLP-1) receptor agonist widely prescribed for type 2 diabetes mellitus and obesity, is generally well tolerated but can be associated with gastrointestinal adverse effects and, rarely, pancreatitis. As its use grows, clinicians must remain aware of potential complications, particularly in patients with additional risk factors.

We report the case of a 57-year-old woman with diabetes, hypertension, and hyperlipidemia who presented with acute severe epigastric pain and persistent vomiting shortly after consuming a large, fatty meal. She had been receiving semaglutide for several months. Laboratory studies showed marked leukocytosis, hyperglycemia with an anion gap metabolic acidosis, and a lipase level exceeding 3000 U/L. CT imaging demonstrated acute interstitial edematous pancreatitis with peripancreatic fluid and early concern for necrosis. She was treated conservatively with aggressive intravenous hydration, bowel rest, electrolyte repletion, and analgesia. Her condition improved with supportive care, and she was discharged with close outpatient follow-up. This case highlights the importance of recognizing pancreatitis as a possible multifactorial complication in patients using GLP-1 receptor agonists.

This case highlights the need for clinicians to maintain awareness of pancreatitis as a potential adverse effect in patients receiving semaglutide, especially those with additional risk factors such as biliary pathology or dietary triggers. As the use of GLP-1 receptor agonists continues to grow, careful assessment of abdominal symptoms and early recognition of pancreatic inflammation are essential for optimizing outcomes and guiding safe prescribing practices.

## Introduction

Semaglutide (Ozempic) is a glucagon-like peptide-1 (GLP-1) receptor agonist increasingly used for the treatment of type 2 diabetes mellitus and obesity. Under normal physiologic conditions, GLP-1 is released in response to food intake and is rapidly degraded by dipeptidyl peptidase-4 (DPP-4). Semaglutide mimics endogenous GLP-1 while remaining resistant to DPP-4 degradation, thereby prolonging its therapeutic effects [1,2]. These actions enhance glucose-dependent insulin secretion, suppress glucagon release, delay gastric emptying, and promote satiety, resulting in improved glycemic control and weight loss [2].

Although semaglutide has demonstrated metabolic benefits and favorable cardiovascular outcomes [3], GLP-1 receptor agonists are associated with gastrointestinal adverse effects, including nausea, vomiting, and delayed gastric motility. Additionally, reports have suggested a potential association between GLP-1 receptor agonist therapy and the development of acute pancreatitis [4]. While this adverse effect remains rare, several cases of pancreatitis have been reported in patients receiving semaglutide [5-7]. We present a case of acute pancreatitis following semaglutide therapy to further contribute to the existing literature.

## Case presentation

A 57-year-old woman with a history of type 2 diabetes mellitus treated with insulin glargine 10 units nightly, semaglutide 2 mg once weekly (started in late 2024), glimepiride 1 mg daily, and metformin 500 mg twice daily, as well as hypertension managed with lisinopril 20 mg and hyperlipidemia treated with atorvastatin 40 mg, presented to the emergency department with one day of severe epigastric abdominal pain and repeated episodes of non-bloody, non-bilious vomiting. Her symptoms began after breaking her fast (Iftar) with a heavy, fatty meal the evening prior and progressively worsened overnight, prompting her family to call emergency medical services. She reported a hospitalization one month earlier for abdominal pain that was attributed to possible gallbladder disease, although no gallstones were identified at that time. The patient denies any history of alcohol intake. 

On arrival, the patient appeared pale and mildly dehydrated but was not in acute distress. Vital signs showed a blood pressure of 164/66 mmHg, a heart rate of 93 beats per minute, a respiratory rate of 18 breaths per minute, a temperature of 37.1°C, and an oxygen saturation of 95% on room air. Abdominal examination revealed a soft, non-distended abdomen without tenderness, guarding, or rebound. Cardiopulmonary examination was unremarkable, and there was no peripheral edema.

Initial laboratory studies demonstrated marked leukocytosis (white blood cell count 25.9 × 10³/µL; 71.3% neutrophils) and significant hyperglycemia (glucose 394 mg/dL). The patient was found to have an anion gap metabolic acidosis (but serum ketones were negative) and a markedly elevated serum lipase level greater than 3,000 U/L. Liver function tests showed transaminitis, with an aspartate aminotransferase level of 128 U/L, an alanine aminotransferase level of 284 U/L, and an alkaline phosphatase level of 118 U/L (Table 1).

**Table 1:** Key laboratory findings. AST: aspartate aminotransferase; ALT: alanine aminotransferase; ALP: alkaline phosphatase; WBC: white blood cell

Laboratory test	Result	Normal range (adult)
WBC count	25.9 × 10³/µL	4.0-11.0 × 10³/µL
Neutrophils	71.3%	40-75% of WBCs
Glucose	394 mg/dL	Fasting: 70-99 mg/dL; random: ≤125 mg/dL
Anion gap	Elevated	~8-12 mEq/L (lab dependent)
Lipase	>3000 U/L	~10-140 U/L (assay dependent)
AST	128 U/L	~10-40 U/L
ALT	284 U/L	~7-56 U/L
ALP	118 U/L	~44-147 U/L

Arterial pH and bicarbonate were not in the diabetic ketoacidosis (DKA) range, and serum ketones were negative, so despite marked hyperglycemia and an anion gap acidosis, DKA or starvation ketosis was unlikely, and the acidosis was attributed to pancreatitis‑related stress and volume depletion.

**Figure 1:**
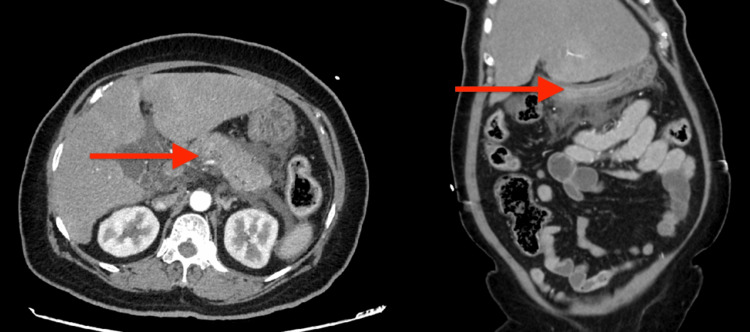
CTA showing acute interstitial edematous pancreatitis with pancreatic edema, peripancreatic fluid, and possible evolving necrosis at the neck. CTA: CT angiogram

A contrast-enhanced CT scan of the abdomen and pelvis showed swelling of the pancreas with surrounding fluid, consistent with acute interstitial edematous pancreatitis, with concern for early necrosis involving the pancreatic neck. Additional findings included a mild reactive ileus, features suggestive of hepatic fibrosis or cirrhosis, and mild retroperitoneal lymphadenopathy (Figure 1).

The patient was admitted with a working diagnosis of acute pancreatitis. A drug-related etiology was considered, while biliary sludge or microlithiasis and idiopathic causes were also included in the differential. Supportive care was initiated, including intravenous fluids, bowel rest, and pain control with intravenous morphine and hydromorphone, along with proton pump inhibitor therapy and antiemetics. Broad-spectrum antibiotics (piperacillin/tazobactam) were administered in the emergency department but were discontinued after admission due to the absence of fever and the low suspicion for an infectious process, with leukocytosis thought to be related to dehydration. Over the following days, liver enzymes and bilirubin levels gradually improved, although mild hypoalbuminemia (3 g/dL) persisted. Electrolyte abnormalities included hyponatremia (133 mmol/L) and hypocalcemia (7.3 mg/dL).

During her hospital stay, the patient's symptoms improved with conservative management, including NPO status, intravenous hydration, and analgesia. Given the low concern for infection, antibiotics were withheld with close monitoring of temperature trends and leukocytosis. A repeat CT scan of the abdomen and a right upper quadrant ultrasound were planned for further evaluation; however, these were not completed before discharge because the patient's symptoms improved and she elected to continue workup as an outpatient. Additional laboratory testing included a lipid panel, viral hepatitis serologies, and an international normalized ratio, prompted by imaging findings suggestive of possible hepatic fibrosis or cirrhosis. As her condition continued to improve, her diet was advanced as tolerated, and her home antihypertensive and antidiabetic medications were resumed. She remained hemodynamically stable throughout hospitalization, with outpatient gastroenterology follow-up arranged for further evaluation of the pancreatitis and liver findings. Semaglutide was permanently discontinued. 

## Discussion

With the increasing popularity of semaglutide and its expanding use in the treatment of obesity, it is important for clinicians to be familiar with its potential adverse effects. One of the most clinically relevant concerns under investigation is acute pancreatitis. Although an association between GLP-1 receptor agonists and pancreatitis has been reported, the underlying relationship remains unclear. Patient comorbidities, as well as medication dose, may influence this risk. Early clinical trials submitted to the Food and Drug Administration (FDA) suggested a higher incidence of pancreatitis compared with placebo; however, more recent studies have reported conflicting findings. The reported risk of pancreatitis in patients receiving GLP-1 receptor agonists appears to vary based on patient characteristics. For example, one study found that patients with type 2 diabetes mellitus and no significant comorbidities had a lower risk of developing pancreatitis when treated with a GLP-1 receptor agonist compared with those not receiving this therapy [8]. In contrast, another study suggested a dose-dependent association between GLP-1 receptor agonist use and the incidence of pancreatitis [9]. These potential safety concerns must be weighed against the expanding clinical indications and therapeutic benefits of this drug class.

Despite these concerns, GLP-1 receptor agonists continue to be widely used due to their well-established efficacy. Their growing role is reflected in current clinical guidelines. The FDA recommends GLP-1 receptor agonists in patients with type 2 diabetes mellitus when metformin is contraindicated, when hemoglobin A1C remains more than 1.5% above target, or when glycemic control is not achieved after three months of therapy [10]. Although initially approved for the management of type 2 diabetes mellitus, GLP-1 receptor agonists have more recently been approved for the treatment of obesity [9]. The FDA currently supports their use in obese patients with comorbidities, particularly for weight reduction and cardiovascular risk reduction [10,11]. In addition, current guidelines recommend prioritizing GLP-1 receptor agonists in patients with atherosclerotic cardiovascular disease, heart failure, or chronic kidney disease. Emerging evidence also suggests a potential role in slowing the progression of chronic kidney disease [10]. Ongoing research continues to explore additional indications for this medication class. As a result of these benefits, GLP-1 receptor agonists have seen a substantial rise in use in recent years, although their high cost and evolving safety profile remain important considerations.

This case is distinctive because pancreatitis developed soon after a large high‑fat Iftar meal in a fasting patient with biliary sludge who was receiving semaglutide, suggesting a multifactorial process rather than an isolated drug reaction. Prolonged fasting can promote bile stasis and sludge formation, while refeeding with heavy fatty meals increases gallbladder activity and may precipitate biliary events in susceptible individuals [12]. Biliary sludge and microlithiasis are now recognized as important causes of so‑called idiopathic acute pancreatitis. GLP‑1 receptor agonists, particularly semaglutide, have been associated with an increased risk of gallbladder‑related adverse events, likely via impaired gallbladder motility and altered bile acid homeostasis.

Given these factors, caution may be warranted when prescribing GLP-1 receptor agonists to patients with a history of gallbladder disease or pancreatitis, and patients should be counseled regarding symptoms that warrant prompt medical attention. While these agents offer significant metabolic and cardiovascular benefits, continued vigilance for rare adverse events such as pancreatitis remains essential. Applying formal causality frameworks, this presentation would be classified as "possible" on the World Health Organization-Uppsala Monitoring Centre (WHO-UMC) scale, and in the possible probable range by the Naranjo algorithm, given the temporal relationship to semaglutide, clinical improvement after discontinuation, and the presence of alternative risk factors such as biliary sludge and a large high‑fat meal. These findings support semaglutide as a contributing factor within a multifactorial etiology rather than a sole cause. This case underscores the importance of maintaining a broad differential diagnosis when evaluating abdominal pain in patients receiving semaglutide and adds to the growing body of literature examining this potential association. Further prospective studies are needed to better define causality and clarify the underlying pathophysiologic mechanisms.

## Conclusions

This case highlights the importance of maintaining awareness of acute pancreatitis as a potential adverse effect of semaglutide. Although GLP-1 receptor agonists provide substantial therapeutic benefits, clinicians should remain attentive to patient-specific risk factors and monitor for early warning signs. Importantly, in patients receiving GLP-1 receptor agonists, pancreatitis should not be attributed to the medication in isolation; a systematic evaluation for other contributing factors, such as biliary disease, large high-fat meals, rapid weight loss, or prolonged fasting, is essential.

In patients with known or suspected gallbladder disease who are using semaglutide while fasting, such as during Ramadan, targeted counseling regarding Iftar meal size and composition may be beneficial. In addition, a low threshold should be maintained for abdominal imaging and serum lipase testing when new epigastric pain develops. As the use of GLP-1 receptor agonists continues to expand, early recognition and prompt evaluation of abdominal symptoms become increasingly important. Further research is needed to better define the true risk of pancreatitis and to clarify the underlying mechanisms involved.

## References

[1] Marso SP, Bain SC, Consoli A (2016). Semaglutide and cardiovascular outcomes in patients with type 2 diabetes. N Engl J Med.

[2] Singh S, Chang HY, Richards TM, Weiner JP, Clark JM, Segal JB (2013). Glucagonlike peptide 1-based therapies and risk of hospitalization for acute pancreatitis in type 2 diabetes mellitus: a population-based matched case-control study. JAMA Intern Med.

[3] Dagher C, Jailani M, Akiki M, Siddique T, Saleh Z, Nadler E (2024). Semaglutide-induced acute pancreatitis leading to death after four years of use. Cureus.

[4] Hughes K, Sumaruth YR, Mohammed E, Sant Bakshsingh V (2024). Acute pancreatitis likely due to semaglutide. Cureus.

[5] Patel F, Gan A, Chang K, Vega KJ (2023). Acute pancreatitis in a patient taking semaglutide. Cureus.

[6] Ayoub M, Chela H, Amin N, Hunter R, Anwar J, Tahan V, Daglilar E (2025). Pancreatitis risk associated with GLP-1 receptor agonists, considered as a single class, in a comorbidity-free subgroup of type 2 diabetes patients in the United States: a propensity score-matched analysis. J Clin Med.

[7] Gu JH, Samarneh M (2025). Dose-dependent pancreatitis risk associated with GLP-1 agonists. J Diabetes Metab Disord.

[8] Collins L, Costello RA (2024). Glucagon-like peptide-1 receptor agonists. StatPearls.

[9] Wilding JP, Batterham RL, Calanna S (2021). Once-weekly semaglutide in adults with overweight or obesity. N Engl J Med.

[10] de Boer IH, Khunti K, Sadusky T (2022). Diabetes management in chronic kidney disease: a consensus report by the American Diabetes Association (ADA) and Kidney Disease: Improving Global Outcomes (KDIGO). Diabetes Care.

[11] (2025). Pharmacologic approaches to glycemic treatment: standards of care in diabetes-2025. Diabetes Care.

[12] Payza U, Kayalı A, Karakaya Z (2021). Effects of Ramadan fasting on gastrointestinal system. J Clin Pract Res.

